# The Inhibition Activity of Natural Methoxyflavonoid from *Inula britannica* on Soluble Epoxide Hydrolase and NO Production in RAW264.7 Cells

**DOI:** 10.3390/ijms25084357

**Published:** 2024-04-15

**Authors:** Jang Hoon Kim, Kyung-Sook Han, Eun-Song Lee, Yong-Goo Kim, Yong-Il Kim, Byoung Ok Cho, Ik Soo Lee

**Affiliations:** 1 Department of Herbal Crop Research, National Institute of Horticultural & Herbal Science, RDA, Chungbuk 27709, Republic of Korea; jhkim53@korea.kr (J.H.K.); kshan9@korea.kr (K.-S.H.); eslee@korea.kr (E.-S.L.); ygkimsy@korea.kr (Y.-G.K.); k007kyi@korea.kr (Y.-I.K.); 2 Institute of Health Science, Jeonju University, 303 Cheonjam-ro, Jeonju-si 55069, Republic of Korea; 3 Km Covergence Research Division, Korea Institute of Oriental Medicine, Daejeon 34054, Republic of Korea

**Keywords:** *Inula britannica*, soluble epoxide hydrolase, flavonoids, NO production

## Abstract

Soluble epoxide hydrolase (sEH) is an enzyme targeted for the treatment of inflammation and cardiovascular diseases. Activated inflammatory cells produce nitric oxide (NO), which induces oxidative stress and exacerbates inflammation. We identify an inhibitor able to suppress sEH and thus NO production. Five flavonoids **1**–**5** isolated from *Inula britannica* flowers were evaluated for their abilities to inhibit sEH with IC_50_ values of 12.1 ± 0.1 to 62.8 ± 1.8 µM and for their effects on enzyme kinetics. A simulation study using computational chemistry was conducted as well. Furthermore, five inhibitors (**1**–**5**) were confirmed to suppress NO levels at 10 µM. The results showed that flavonoids **1**–**5** exhibited inhibitory activity in all tests, with compound **3** exhibiting the most significant efficacy. Thus, in the development of anti-inflammatory inhibitors, compound **3** is a promising natural candidate.

## 1. Introduction

*Inula britannica* L. (Asteraceae), commonly known as British yellowhead or British elecampane, is a perennial herb native to East Asia and parts of Europe [1]. It grows to a height of 1–2 m, has lance-shaped leaves, and blooms bright yellow flowers similar to sunflowers. *I. britannica* is a traditional medicinal plant that has been used for centuries to treat a variety of ailments, such as indigestion, diarrhea, dysentery, colic, flatulence, menstrual cramps, respiratory problems, and skin problems [1,2]. The pharmacological activities of the plant include anti-inflammatory, antimicrobial, antiviral, antioxidant, hepatoprotective, and gastroprotective activities [1,2,3]. These properties have been attributed to the presence of diverse secondary metabolites within the plant. The main chemical constituents of *I. britannica* are sesquiterpene lactones, flavonoids, tannins, saponins, polysaccharides, volatile oils, and steroids [1,2,3]. Sesquiterpene lactones are the most abundant active constituents, and they are thought to be responsible for the plant’s anti-inflammatory, anticancer, and antibacterial properties [4]. Flavonoids are also among the main bioactive components of *I. britannica* and their enzyme inhibitory, antioxidant, and cytotoxic properties have been described [1,5].

Soluble epoxide hydrolase(sEH) is an α/β hydrolase fold protein expressed in the cytosol and peroxisome of mammalian cells [6]. The enzyme consists of two 62.4 kDa monomers. The 34 kDa C-terminal hydrolase domain converts epoxyeicosatrienoic acids (EETs) into dihydroxyeicosatrienoic acids (DHETs) [6,7]. EETs are produced through the epoxidation of arachidonic acid by epoxygenase CYP enzymes [8]. They include four regioisomeric EETs: 5,6-, 8,9-, 11,12-, and 14,15-EETs [8]. EETs suppress cytokine-induced inflammatory responses, such as those mediated by NF-kB and IkB kinase [6]; 11,12-EET has shown promise as an endothelium-derived hyperpolarizing factor in the coronary vascular system [9]. Recently, an sEH inhibitor effective for lowering blood pressure in mice with angiotensin-II-induced hypertension was described [10]. This inhibitor, the urea pharmacophore 12-(3-adamantan-1-yl-ureido) dodecanoic acid (AUDA), is a combination of adamantane and fatty acid chains and one of the most potent inhibitors of sEH [11]. However, a disadvantage in its further development is its rapid metabolism in vivo [11]. Recently, sEH inhibitors have been developed from natural plants; for example, 3β-hydroxy-25-anhydro-alisol F, 3β-hydroxy-alisol G [12], cannabisin B, grossamide [13], 4H-tomentosin, xanthalongin, and linoleic acid [14] were discovered as representative inhibitors. Nitric oxide (NO) is produced by the reaction of L-alanin and O_2_ by three NO synthase (NOS) isoforms, which are nNOS derived from neuron, eNOS from endothelial tissue and iNOS [15]. nNOS and eNOS produce small amounts of NO in response to the stimulation of receptors in neurons and endothelial tissues, respectively [15]. On the other hand, iNOS produces large amounts of NO in response to inflammatory reactions [15]. LPS- and IFN-gamma-stimulated murine macrophages produce NO by iNOS. This is known to induce apoptosis of inflammatory cells [16].

In ongoing efforts to discover effective natural sEH inhibitors for therapeutic applications, we isolated five flavonoids from an extract of *I. britannica* flowers. These compounds were evaluated for their ability to inhibit sEH and NO production. Their ligand–receptor interaction mechanisms were also investigated through enzyme kinetics and computational chemistry studies. This report describes the isolation and sEH inhibitory effects of the five isolated flavonoids.

## 2. Results

### 2.1. Isolation and Identification of Compounds 1–5

**Figure 1:**
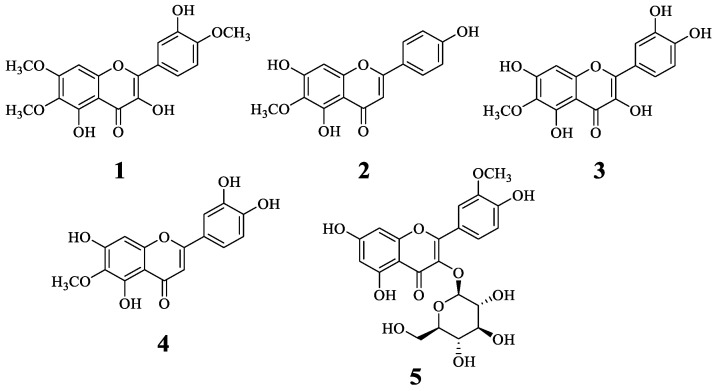
The structure of compounds isolated from *I. britannica* flowers.

Five compounds were isolated from the ethanol extract of *I. britannica* flowers through a series of chromatographic separation steps based on sEH inhibitory activity. Compounds **1**–**5** were obtained as yellowish amorphous powders and exhibited two characteristic UV absorption maxima, around 338–355 and 254–272 nm, corresponding to bands I and II of the A-ring and B-ring of flavonoids, respectively [17]. Through a detailed comparison with the ^1^H- and ^13^C-NMR data from the literature, the following five flavonoids were identified: eupatin (**1**) [5], hispidulin (**2**) [18], patuletin (**3**) [18], nepetin (**4**) [19], and isorhametin-3-*O*-glucoside (**5**) [20] (Figure 1).

### 2.2. Inhibitory Activity of Compounds **1**–**5** on sEH

**Figure 2:**
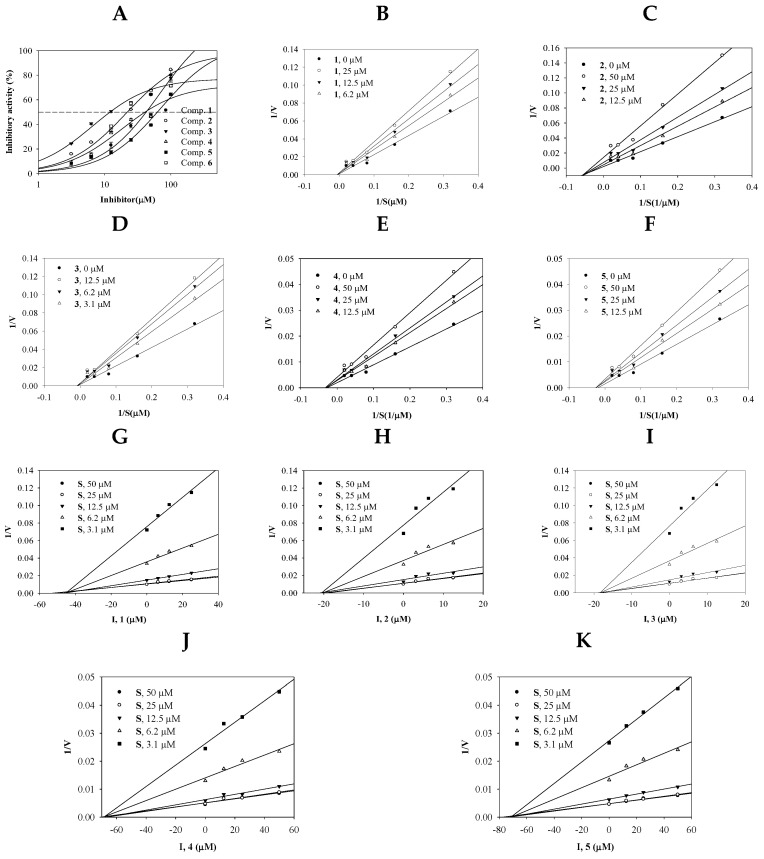
The inhibitory activity (**A**), Lineweaver–Burk (**B**–**F**) and Dixon (**G**–**K**) plots of compounds on sEH.

The ability of isolated compounds **1**–**5** to inhibit sEH was evaluated. The rate of inhibitory activity was calculated using Equation 1. As shown in Figure 2A and Table 1, the sEH inhibitory activity of the five flavonoids was dose-dependent over a concentration range of 3.1 to 100 µM. The IC_50_ values of compounds **1**–**5** when tested with sEH was determined using Equation 2. Based on tests of six different concentrations of the five compounds, the IC_50_ values were 42.6 ± 0.8, 22.2 ± 0.3, 12.1 ± 0.1, 40.9 ± 0.6, and 62.8 ± 1.8 µM, respectively.

Determinations of enzyme kinetics yield insights into the interactions between inhibitors and enzymes. In such studies, the changes in the initial velocity (*v_0_*) of the enzymatic reaction induced by the inhibitor depending on the substrate concentration are monitored [21]. To investigate the effects of the five flavonoids (**1**–**5**) on sEH, a substrate concentration of 3.1–50 µM was used. As shown in Figure 2B–F and Table 1, compounds **1**–**5** suppressed the catalytic reaction of sEH by acting as noncompetitive inhibitors. The *k*_i_ values calculated from the Dixon plot were 43.9, 19.8, 17.5, 63.5, and 68.7 µM, respectively (Figure 2G–K).

### 2.3. Static Molecular Modeling between Compounds **1**–**5** and sEH

**Figure 3:**
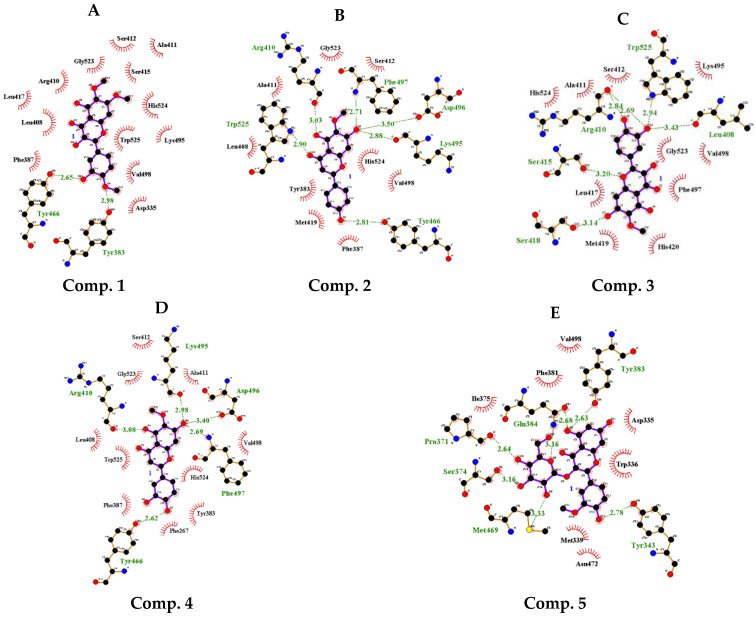
The best docking pose and hydrogen bonds of compounds **1**−**5** with sEH (**A**–**E**).

The binding mode of the five compounds, as noncompetitive inhibitors, with the enzyme was calculated using a blind docking method. Compounds **1**–**5** had Autodock scores of −8.16, −8.34, −8.59, −8.18, and −7.53 kcal/mol, respectively. Compound **1** formed hydrogen bonds with Tyr383(2.98 Å) and Tyr466 (2.65 Å); compound **2** interacted with residues Arg410(3.03 Å), Trp466(2.81 Å), Lys495(2.88 Å), Asp496(3.50 Å), Phe497(2.71 Å), and Trp525(2.90 Å); compound **3** specifically bonded with Leu408(3.43 Å), Arg410(2.68, 2.84 Å), Ser415(3.20 Å), Ser418(3.14 Å), and Trp525(2.94 Å); compound 4 interacted with Arg410(3.08 Å), Tyr466(2.62 Å), Lys495(2.98 Å), Asp496(3.40 Å), and Phe497(2.69 Å); and compound **5** interacted with Tyr343(2.78 Å), Pro371(2.64 Å), Ser374(3.16 Å), Tyr383(2.63 Å), Gln384(2.68, 3.16 Å), and Met469(3.33 Å) (Figure 3A–E and Table 2).

### 2.4. Dynamic Molecular Modeling between Compound 3 and sEH

**Figure 4:**
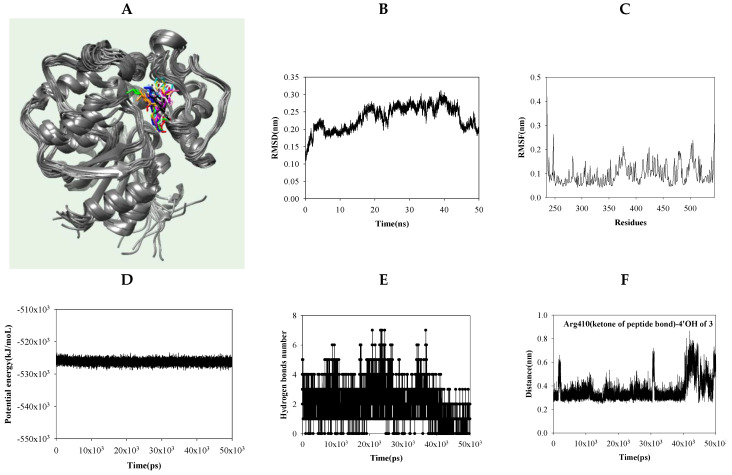

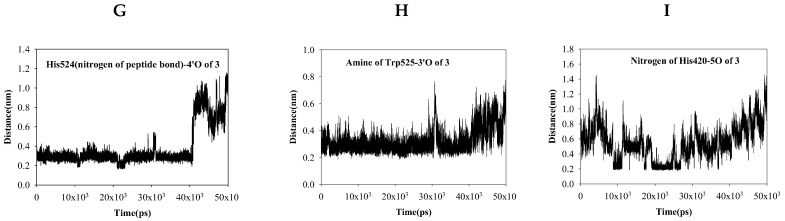
The overlapped pose of inhibitor **3** (**A**) with sEH for 30 ns (red: 0 ns, orange: 5 ns, yellow: 10 ns, green: 15 ns, cyan: 20 ns, blue: 25 ns, conflower blue: 30 ns, purple: 35 ns, menganta: 40 ns, white: 45 ns, black: 50 ns). The RMSD (**B**), RMSF (**C**), potential energy (**D**), and hydrogen bonds (**E**) of the simulation. The distance of key amino acids from inhibitor **3** (**F**–**I**).

Compound **3** had the lowest IC_50_ value and docking score. During molecular dynamics simulations (Figure 4A) conducted at a potential energy −5.3 × 10^5^ kcal/mol, compound **3** had root mean square deviation (RMSD) and root mean square fluctuation (RMSF) values of 0.30 nm and 0.2 nm, respectively (Figure 4B–D). During this simulation, **1**–**4** hydrogen bonds formed between compound **3** and sEH (Figure 4E). From these values, the stability of the molecular structures, the accuracy of the simulations, as well as the flexibility of the molecules and their structural significance could be assessed. From 0 ns to 40 ns, the 4′-OH and 4′O of compound **3** were approximately 3.5 Å away from the ketone and nitrogen within the peptide bonds of Arg410 and His524, respectively (Figure 4F,G). The NH of TR525 maintained a consistent distance of 3.5 Å from the 3′-O of compound **3** (Figure 4H), while the N of His420 was separated by a distance of 3.5 Å from the 5’-O of compound **3** between 10 ns and 40 ns (Figure 4I).

### 2.5. Inhibition of NO Production by Compounds **1**–**5** in Poly(I:C)-Stimulated RAW264.7 Cells

**Figure 5:**
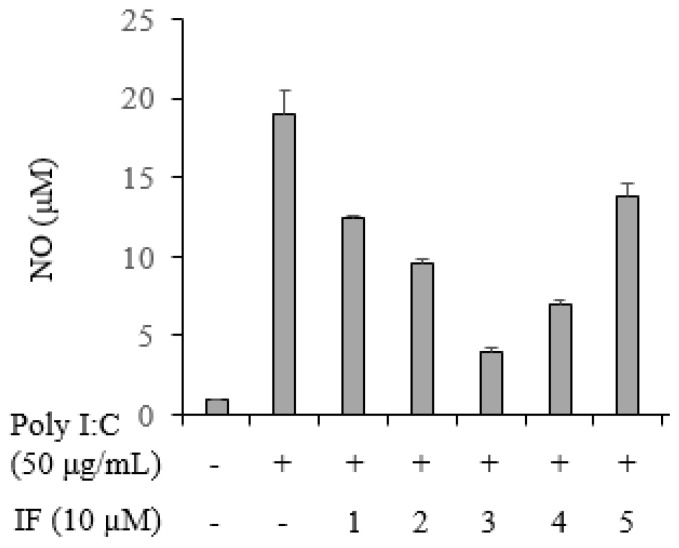
Effects of compounds **1**–**5** on the inhibition of NO production.

The activity of compounds **1**–**5** with respect to NO production by poly(I:C)-induced RAW264.7 cells was evaluated by measuring the amount of nitrite that accumulated in the culture medium using the Griess method. When tested at a concentration 10 µM, none of the compounds affected the viability of RAW264.7 cells. In unstimulated RAW264.7 cells, NO production was ~ 0.91 µM, whereas in poly(I:C)-induced cells, it was 19.0 ± 1.5 µM. In induced cells simultaneously treated with compounds **1**–**5** at a concentration of 10 µM, NO production was 12.5 ± 0.1, 9.6 ± 0.2, 4.0 ± 0.2, 7.0 ± 0.3, and 13.9 ± 0.8 µM, respectively (Figure 5).

## 3. Discussion

Compounds derived from medicinal plants and exhibiting inhibitory activity against sEH have received considerable attention because of their potential therapeutic applications. These compounds primarily include natural flavonoids, terpenoids, alkaloids, anthraquinones, stilbenes, and phenolics [6,22,23,24,25,26]. Flavonoids are a class of polyphenolic compounds found abundantly in fruits, vegetables, and medicinal plants. Several flavonoids, such as quercetin and kaempferol, are potent sEH inhibitors [26]; others exhibit anti-inflammatory, antioxidant, and cardioprotective properties [27]. Terpenoids are diverse secondary metabolites produced in many plant species. They include triterpenes and sesquiterpenes, with considerable inhibitory effects on sEH determined for 11-deoxy-25-anhydroalisol E and 11-deoxyalisol B, two protostane-type triterpenoids found in Alisma orientale [28].

The genus Inula is a diverse group of flowering plants in the Asteraceae family, commonly known as the aster, daisy, or sunflower family. Inula species are widely distributed across Europe, Asia, and Africa, with more than 100 recognized species [1]. Many species of Inula have long been used in traditional medicine, including for the treatment of respiratory conditions, digestive issues, and skin disorders [1,2,4]. Among the compounds that contribute to the medicinal properties of Inula species are sesquiterpenes, flavonoids, essential oils, polysaccharides, and other secondary metabolites [1,2,3]. The specific compounds and their concentrations vary depending on the species. *I. britannica*, also known as British yellowhead, is one the most prominent medicinal plants of the genus Inula. Because of its anti-inflammatory, antibacterial, and antifungal properties, it has been used in traditional Chinese medicine [1,2], such as for the treatment of inflammatory conditions, digestive disorders, and skin ailments [1,2]. Flavonoids and sesquiterpene lactones are among the primary active ingredients believed to be responsible for the medicinal effects of *I. britannica* [1,2]. Thus, to identify potent sEH inhibitors from medicinal plants, we focused on the flavonoids in *I. britannica* flowers. This 50% ethanolic extract improved the relative Gr-1 density and MPO of tissue in acute lung injury. Also, the concentration of 8,9-EET, 11,12-EET, and 14,15-EET were increased as the extract of *I. japonica* inhibited recombinant sEH [29]. This effect downregulated the MAPK and NF-kB signaling pathways and oxidative stress and, on the other hand, upregulated the Nrf2 signaling pathway [29]. This extract was determined to be quercetin, quercetin-3-*O*-glucopyranoside, quercetin-3-*O*-rhamnopyranoside, luteolin, and kaempferol [25]. Three flavone types, including chrysoeriol, luteolin-7-*O*-glucopyranoside, and isorhamnetin-7-*O*-glucopyranoside, were reported as sEH non-competitive inhibitors with IC_50_ values of ~38(~11 µg/mL), ~32(~14 µg/mL), and ~68(~42 µg/mL) µM, respectively [23]. Flavonoles and flavone-3-*O*-glycosides also had inhibitory activity with IC_50_ values within ~22.5 to 61.9 µM [26]. Methoxyl-flavone was found to have an IC_50_ of ~15 µM [30]. On the other hand, flavonone had a very low activity with an IC_50_ of ~250 µM [31]. Five flavonoids (eupatin, hispidulin, patuletin, nepetin, and isorhametin-3-*O*-glucoside) were isolated (**1**–**5**) and subsequently shown to be noncompetitive inhibitors of sEH, with *k*_i_ values of 43.9, 19.8, 17.5, 63.5, and 68.7 µM, and IC_50_ values against sEH of 42.6 ± 0.8, 22.2 ± 0.3, 12.1 ± 0.1, 40.9 ± 0.6, and 62.8 ± 1.8 µM, respectively. All the compounds were methoxyl-flavone. Moreover, the other methoxyflavonoids and lignans from this plant also showed sEH inhibitory effects similar to the results of this experiment [32].

Overall, flavones and flavonols tended to have relatively higher efficacy than that of flavonones. Their values obtained from the molecular docking study were −8.16, −8.34, −8.59, −8.18, and −7.53 kcal/mol, and those determined for the inhibition of NO production from poly(I.C.) stimulated RAW264.7 cells were 12.5 ± 0.1, 9.6 ± 0.2, 4.0 ± 0.2, 7.0 ± 0.3, and 13.9 ± 0.8 µM, respectively. The results of the enzyme assays, enzyme kinetics, computer simulations, and NO experiments were consistent. NOS biosynthesize hydrogen peroxide, superoxide, and NO. When the L-arginine concentration is low, the generated superoxide reacts with NO, producing peroxynitrite and adversely affecting cell survival [33]. These nitrogen species are related to inflammation and have been implicated in a variety of diseases, such as cancer, coronary heart disease, and rheumatoid arthritis [34]. The potential inhibitor **3** was reported to decrease TNF-α and IL-1β levels in LPS-activated THP-1 cells and arthritic rats [35].

Furthermore, compound **3**, which exhibited the most pronounced effects among the five compounds, was meticulously examined for its binding in flexible enzyme states. When examined by rigid X-ray crystallography, compound **3** formed hydrogen bonds with Arg410, Tyr466, Lys495, Asp496, Phe497, and Trp525, as observed in the simulations. However, when simulated in a flexible state, it maintained distances conducive to hydrogen bonding with the ketone and nitrogen of the peptide bond bearing Arg410 and His524, as well as with the amine and nitrogen of Trp525 and His420, respectively. The catalytic triads of rat sEH were revealed to be Asp333, Asp495, and His523. Asp333, which is nucleophile, attacks a carbon at the epoxide of substrate. The imidazole of His523 paired with Asp495 binds to hydrogen of water. The oxygen of water attacks the ketone of Asp333, which is ester-bound to the substrate, and then the substrate is hydrolyzed [36]. Those of human sEH were Asp335, As496, and His524 [37]. Among these, it was confirmed that compound **3** can directly affect His524 during the MD process.

The binding of compound **3** to the enzyme complex occurs in a fluid state. Molecular dynamics, based on the molecular docking results, indicated a higher probability of amino acids in the presence of inhibitor **3**. Trp525 and His420, rather than Arg410 and His524, were identified as the key amino acid residues involved in inhibitor binding.

## 4. Materials and Methods

### 4.1. General Experimental Procedures

^1^H (400 MHz) and ^13^C (100 MHz) nuclear magnetic resonance (NMR) spectra were obtained using a Bruker DRX-400 spectrometer (Bruker, Billerica, MA, USA) with tetramethylsilane as the internal standard. Thin-layer chromatography (TLC) was performed on pre-coated silica gel 60 F254 (0.25 mm; Merck, Rahway, NJ, USA) and reversed-phase-18 F254s plates (0.25 mm; Merck). Column chromatography was performed using a silica gel (60 A, 70–230, or 230–400 mesh ASTM; Merck, Germany) and reversed-phase silica gel (ODS-A, S-75 μm; YMC, Kyoto, Japan). Spots were detected by UV light (254 nm) after spraying the plates with 10% H_2_SO_4_ followed by heating. Tris (catalog no. B9754) and bovine serum albumin (BSA, A8806) were purchased from Sigma-Aldrich (St. Louis, MO, USA), and human recombinant sEH (10011669), 3-phenyl-cyano(6-methoxy-2-naphthalenyl)methyl ester-2-oxiraneacetic acid (PHOME, 10009134), and AUDA (10007972) were purchased from Cayman Chemical (Ann Arbor, MI, USA). Greiss reagent and lipopolysaccharide (LPS) were purchased from Sigma-Aldrich (St. Louis, MO, USA). Dulbecco’s modified Eagle medium (DMEM) and fetal bovine serum were purchased from Gibco (Grand Island, NY, USA). Penicillin/streptomycin antibiotics came from Invitrogen (Carlsbad, CA, USA).

### 4.2. Plant Material

Flowers of *I*. *britannica* were purchased from a traditional herbal medicine store in Daejeon, Republic of Korea, in August 2020 and authenticated by one of the authors (ISL). A voucher specimen (IJ2020–032) was archived in the herbarium of the Korea Institute of Oriental Medicine, Republic of Korea.

### 4.3. Extraction and Isolation

Air-dried flowers of *I*. *britannica* (400 g) were extracted three times with ethanol. The resulting extracts were filtered and concentrated (30 g) and then subjected to silica gel column chromatography with a methylene chloride–methanol gradient solvent system. Three fractions (A–C) based on the TLC patterns were obtained. Fraction A was further chromatographed on a silica gel column using a methylene chloride–methanol gradient solvent system to yield three subfractions (A1–A3). Fraction A2 was purified over a reversed-phase silica gel column and eluted with a methanol–water gradient solvent system to obtain compounds **1** (15 mg), **2** (25 mg), and **3** (22 mg). Fraction B was subjected to silica gel column chromatography with a methylene chloride–methanol gradient solvent system, yielding four subfractions (B1–B4). Fraction B2 was further purified on a reversed-phase silica gel column and eluted with a methanol–water gradient solvent system to afford compounds **4** (23 mg) and **5** (32 mg). Fraction C was chromatographed on a silica gel column using a methylene chloride–methanol gradient solvent system to generate two subfractions (C1 and C2).

### 4.4. sEH Activity Assay

In 96-well plates, 20 µL of isolated compounds **15** in methanol was mixed with 100 µL of 25 mM Tris–HCl (pH 7.0) buffer containing 0.1% BSA, followed by adding 30 µL of 1.0 µg/mL sEH and then 50 µL of 40 μM PHOME. The reaction product was measured using a fluorescent photometer (excitation filter: 330 nm, emission filter: 465 nm).

Inhibitor efficiency (%) was calculated as shown in Equation (1):

Inhibitory activity = 100 − [(I4_0_ − I_0_)/(M_40_ − M_0_)] × 100 (1)

where I_40_ and M_40_ are the fluorescence of the candidate inhibitor (I) and methanol (M) after 40 min, and I_0_ and M_0_ are the fluorescence of I and M at 0 min, respectively.

The IC_50_ values were calculated using Equation (2):

y = y_0_ + [(a × x)/(b + x)] (2)

where y_0_ is the minimum value on the y-axis, a represents the disparity between the maximum and minimum values, and b is the x value corresponding to 50%.

### 4.5. Molecular Docking

Three-dimensional (3D) structures of the candidate inhibitors were generated and optimized using Chem3D Pro (CambridgeSoft, Cambridge, MA, USA). The 3D structure of sEH (ID: 3ANS) was retrieved from the RCSB Protein Data Bank. The enzyme’s structure was modeled by excluding water and 4-cyano-*N*-[(1*S*,2*R*)-2-phenylcyclopropyl]benzamide, followed by hydrogen addition, using AutoDockTools 4.2 (Scripps Research, La Jolla, CA, USA) with the Gasteiger charge model. Flexible patuletin docking employed a torsion tree approach to identify the torsion root and rotatable bonds. Docking was conducted within a grid box size of 126 × 126 × 126 with 0.375 Å spacing to accommodate a non-competitive inhibitor within the enzyme. Molecular docking was carried out based on a Lamarckian genetic algorithm using Chimera ver. 1.14 (University of California, San Francisco, San Francisco, CA, USA). In the visualization and analysis of the results, LigPlot 2.2 (European Bioinformatics Institute, Hinxton, UK) was used.

### 4.6. Molecular Dynamics

Simulation of the sEH-patuletin (**3**) complex was performed using Gromacs 4.6.5, employing the CHARMM all-atom force field to charge the complex. The ligand structure, generated initially as an str file by the GGenFF server, was converted into gro and itp files using CHARMM36-ff. Subsequently, the charged sEH-patuletin (**3**) complex was solvated in a cubic water box using the simple point charge water model and ionized with sodium ions. mdp files of ions, energy minimization, NVT, NPT, and MD were generated according to GROMACS instructions and used for energy minimization, employing the steepest-descent method until a maximal force of 10 kJ/mol was reached. The equilibration phase involved NVT (canonical ensemble) at 300 K for 100 ps, followed by NPT (isothermal–isobaric ensemble) at 1 bar for another 100 ps. Finally, a molecular dynamics simulation was conducted for 50 ns. The results were analyzed using g utility and visualized using SigmaPlot 15 (San Jose, CA, USA) and Chimera 1.17.3 (San Francisco, CA, USA).

### 4.7. Cell Culture and Viability

RAW264.7 macrophages in DMEM containing 10% FBS and 1% penicillin/streptomycin were seeded into a 96-well plate at a concentration of 2 × 10^5^ cells/well and then cultured in a CO_2_ incubator. After 24 h, 10 µM of the candidate inhibitor was added. After another 24 h incubation, 10 µL water-soluble tetrazolium salt (WST-8) was added to each well and the plate was incubated for 4 h. The absorbance at 450 nm was measured using a spectrophotometer (Tecan Group, Ltd., Mannedorf, Switzerland).

### 4.8. Nitric Oxide Assay

RAW264.7 macrophages were initially cultured as described above and then pretreated for 2 h with 10 µM of the candidate inhibitor. Then, they were stimulated with 1 µg LPS /mL for 24 h. Then, 100 µL each of supernatant and Griess reagent were added to the plate. The absorbance at 450 nm was measured as described.

### 4.9. Statistical Analysis

All measurements were performed in triplicate across three independent experiments. The results are expressed as the mean ± standard error of the mean (SEM). The data were analyzed using Sigma Plot 15 (Systat Software Inc., San Jose, CS, USA).

## 5. Conclusions

The ethanol extract of *I. britannica* was purified using column chromatography to obtain five flavonoids **1**–**5**. Their chemical structures, determined spectrophotometrically, revealed them to be eupatin (**1**), hispidulin (**2**), patuletin (**3**), nepetin (**4**), and isorhametin-3-*O*-glucoside (**5**). The IC_50_ values of compounds **1**–**5** when tested against sEH were in the range of 12.1 ± 0.1–62.8 ± 1.8 µM. The IC_50_ value of compound **3** was the lowest at 12.1 ± 0.1 µM. The *k*_i_ values calculated from the Dixon plot were 43.9, 19.8, 17.5, 63.5, and 68.7 µM, and the Autodock scores were −8.16, −8.34, −8.59, −8.18, and −7.53 kcal/mol, respectively. Potential inhibitor **3** was shown to bind very intimately with sEH in a fluid state based on molecular dynamics. In particular, it was confirmed that this maintained a sufficient distance of ~ 3.5Å from Arg410 and Trp525, which participate in hydrogen bonding in the docking. In poly(I:C)-stimulated RAW264.8 cells, compounds **3** and **4** inhibited NO production by more than 50% at 10 µM. In the enzyme assay and in studies of enzyme kinetics, molecular simulation, and NO inhibition, flavonoids **1**–**5** showed similar trends. Our results indicate that compound **3** should be further examined as an anti-inflammatory agent in inhibition experiments involving other cell types and animal models.

## Figures and Tables

**Table 1:** The inhibitory activity of the inhibitors **1**–**5** toward sEH.

	IC_50_ (µM) ^a^	Binding Mode (µM)
**1**	42.6 ± 0.8	non-competitive (43.9)
**2**	22.2 ± 0.3	non-competitive (19.8)
**3**	12.1 ± 0.1	non-competitive (17.5)
**4**	40.9 ± 0.6	non-competitive (63.5)
**5**	62.8 ± 1.8	non-competitive (68.7)
**AUDA ^b^**	12.3 ± 0.3 nM	

^a^ All compounds examined in a set of triplicated experiment. ^b^ Positive control.

**Table 2:** The hydrogen bond interactions and binding score between sEH and the inhibitors.

Inhibitor	Hydrogen Bonds (Å)	Autodock Score (kcal/mol)
**1**	Tyr383(2.98), Tyr466(2.65)	−8.16
**2**	Arg410(3.03), Tyr466(2.81), Lys495(2.88), Asp496(3.50), Phe497(2.71), Trp525(2.90)	−8.34
**3**	Leu408(3.43), Arg410(2.84, 2.69), Ser415(3.20), Ser418(3.14), Trp525(2.94)	−8.59
**4**	Arg410(3.08), Tyr466(2.62), Lys495(2.98), Asp496(3.40), Phe497(2.69),	−8.18
**5**	Tyr343(2.78), Pro371(2.64), Ser374(3.16), Tyr383(2.63), Gln384(3.16), Met469(3.33)	−7.53

## Data Availability

Data are contained within the article.
